# Clinical efficacy and safety of tenecteplase combined with argatroban for acute ischemic stroke: A single-center retrospective cohort study

**DOI:** 10.1097/MD.0000000000049809

**Published:** 2026-07-17

**Authors:** Shaoyue Huang, Xiaoxuan Wang, Dongxu Li, Xiaowei Qiu, Zhen Hong

**Affiliations:** a The Second Department of Cerebrovascular Disease, Cangzhou Central Hospital, Cangzhou City, Hebei Province, China; b Department of Neurophysiology, Cangzhou Central Hospital, Cangzhou City, Hebei Province, China; c Department of Neurovascular Intervention, Cangzhou Central Hospital, Cangzhou City, Hebei Province, China.

**Keywords:** acute ischemic stroke, argatroban, intravenous thrombolysis, modified Rankin Scale, National Institutes of Health Stroke Scale, tenecteplase

## Abstract

Acute ischemic stroke remains a major cause of death and disability, and intravenous thrombolysis is central to early reperfusion; however, post-lysis thrombin activity may contribute to rethrombosis and incomplete reperfusion. We conducted a single-center retrospective cohort study of consecutive adults with acute ischemic stroke admitted between January 2023 and December 2024, comparing a control regimen of standard-dose recombinant tissue plasminogen activator (alteplase 0.9 mg/kg, maximum 90 mg) with an observation regimen of low-dose recombinant tissue plasminogen activator (0.6 mg/kg, maximum 60 mg) plus argatroban initiated after thrombolysis in clinically stable patients. Because treatment strategy was selected in routine care rather than randomly assigned, all comparative findings were interpreted as associations. Among 125 patients (control n = 67; observation n = 58) with comparable baseline characteristics, the observation regimen was associated with greater early neurological improvement on the National Institutes of Health Stroke Scale at 24 hours (median change, 4 vs 3; *P* = .001) and day 7 (6 vs 4; *P* < .001), with higher responder rates (National Institutes of Health Stroke Scale decrease ≥4) at 24 hours (67.2% vs 41.8%; *P* = .004) and day 7 (81.0% vs 59.7%; *P* = .010). Favorable 90-day functional outcomes on the modified Rankin Scale (mRS) were more frequent (mRS 0–1: 69.0% vs 47.8%, *P* = .017; mRS 0–2: 87.9% vs 70.1%, *P* = .016), with a more favorable ordinal mRS distribution (*P* = .001). Recanalization was more frequent (*P* = .028), infarct growth was lower (*P* = .001), and early neurological deterioration was less common (*P* = .037). Symptomatic intracranial hemorrhage and other adverse events were uncommon, without significant between-group differences. These real-world findings should be considered hypothesis-generating and require prospective randomized validation.

## 1. Introduction

Acute ischemic stroke (AIS) remains a leading cause of death and long-term disability worldwide, and early reperfusion therapy is central to limiting infarct growth and improving functional recovery. Intravenous thrombolysis is widely accessible; however, its clinical effectiveness is constrained by the time-dependent decline in benefit, incomplete macrovascular recanalization, heterogeneity in tissue-level reperfusion, and the risk of hemorrhagic transformation.^[1,2]^ Against this background, tenecteplase has emerged as an increasingly adopted thrombolytic agent because it can be administered as a single intravenous bolus and has pharmacologic properties that support efficient fibrinolysis in routine stroke workflows. Recent regulatory and implementation-focused discussions have highlighted the expanding clinical footprint of tenecteplase for AIS thrombolysis, particularly as health systems seek simplification of dosing and reductions in treatment delay.^[3]^

The contemporary evidence base for tenecteplase continues to evolve. In patients with minor ischemic stroke and proven intracranial occlusion, the TEMPO-2 phase 3 trial reported no functional benefit and potential harm with tenecteplase compared with standard care, underscoring that thrombolysis intensification may not be uniformly beneficial in low-deficit populations and that patient selection remains critical.^[4]^ Conversely, in imaging-selected patients treated in an extended time window, perfusion-guided strategies have supported the potential role of tenecteplase beyond conventional onset-based paradigms. A randomized trial in the 4.5- to 24-hour window using perfusion imaging selection reported clinically relevant outcome signals, complementing phase 2 multicenter experience with extended-window tenecteplase protocols.^[5]^ Real-world cohorts have also described the feasibility and workflow advantages of tenecteplase implementation, including time-metric improvements and early neurological response profiles compared with alteplase.^[6,7]^ Despite advances in thrombolytic therapy, thrombin generation and coagulation pathway activation may persist after fibrinolysis and contribute to early rethrombosis, incomplete reperfusion, microvascular obstruction, and early neurological deterioration (END). Adjunctive anticoagulation with a direct thrombin inhibitor is therefore biologically plausible as a strategy to stabilize reperfusion and mitigate thrombus propagation. Argatroban, a parenteral direct thrombin inhibitor with rapid onset and a short half-life, has been evaluated across AIS populations, with systematic reviews emphasizing an acceptable bleeding profile but heterogeneous efficacy signals that appear sensitive to timing, clinical phenotype, and concomitant antithrombotic exposure.^[8,9]^ Emerging observational work further suggests that earlier initiation of argatroban may be associated with improved functional outcomes in selected AIS cohorts, supporting continued investigation into its optimal integration with reperfusion therapy.^[10]^

Accordingly, evaluating thrombolysis combined with argatroban in AIS is clinically relevant because it targets complementary mechanisms – fibrin clot lysis and thrombin-mediated rethrombosis – while also aligning with practical workflow objectives. The present study contributes real-world association data on early neurological improvement, functional recovery, and safety endpoints in patients treated with thrombolysis-based strategies with or without adjunctive argatroban, providing evidence to inform future prospective validation and patient-selection strategies rather than establishing causality.

## 2. Methods

### 2.1. Study design

This study was approved by the Ethics Committee of Cangzhou Central Hospital. This single-center retrospective cohort study consecutively enrolled patients with AIS who were admitted between January 2023 and December 2024. Participants were categorized according to the administered reperfusion strategy into a control group (conventional standard-of-care management plus standard-dose intravenous thrombolysis with recombinant tissue plasminogen activator [rt-PA]) and an observation group (conventional standard-of-care management plus low-dose intravenous rt-PA combined with argatroban). Treatment assignment was not randomized; the administered strategy reflected clinician decision-making and institutional practice in routine care. Therefore, selection bias and confounding by indication were considered important design-related limitations. Eligible patients met the following criteria: age ≥18 years; AIS diagnosed based on typical clinical presentation and confirmed by neuroimaging (computed tomography [CT]/magnetic resonance imaging [MRI]); treatment with intravenous thrombolysis within the guideline-recommended therapeutic window, with complete documentation of key baseline variables (including symptom onset time, baseline National Institutes of Health Stroke Scale [NIHSS] score, and imaging findings); and availability of in-hospital safety and efficacy outcomes. Exclusion criteria were: intracranial hemorrhage (ICH) or nonischemic stroke on baseline imaging; known contraindications to thrombolysis and/or argatroban (including active bleeding, recent major surgery/trauma, suspected aortic dissection, or uncontrolled severe hypertension); severe hepatic dysfunction, severe renal impairment, or other conditions conferring a high bleeding risk; and coagulation abnormalities or recent use of anticoagulants incompatible with thrombolysis per guidelines. The study was conducted in accordance with the Declaration of Helsinki. Because this was a retrospective study, patients’ informed consent was waived.

### 2.2. Thrombolysis and anticoagulation protocols

#### 2.2.1. Control group protocol (standard care + standard-dose intravenous rt-PA)

All patients received guideline-concordant standard-of-care management for AIS, including neurological monitoring, airway/oxygenation support when indicated, blood pressure and glucose control, temperature management, and individualized treatment of vascular risk factors. In addition, intravenous thrombolysis was performed using rt-PA (alteplase) at 0.9 mg/kg (maximum total dose, 90 mg), administered as an initial 10% intravenous bolus followed by infusion of the remaining 90% over 60 minutes, within the recommended therapeutic time window and according to the institutional stroke thrombolysis pathway. Antithrombotic agents were generally deferred during the immediate post-thrombolysis period according to standard practice, and subsequent antiplatelet and statin therapy were initiated when clinically appropriate.

#### 2.2.2. Observation group protocol (standard care + low-dose intravenous rt-PA + argatroban)

Patients received the same standard-of-care management as described above. Intravenous thrombolysis was performed using low-dose rt-PA (alteplase) at 0.6 mg/kg (maximum total dose, 60 mg), administered as an initial 10% intravenous bolus followed by infusion of the remaining 90% over 60 minutes, consistent with the study protocol. Argatroban was then initiated after completion of rt-PA infusion in patients without clinical or radiographic evidence of hemorrhagic complications, and was administered according to institutional anticoagulation procedures with dose titration guided by coagulation monitoring (e.g., activated partial thromboplastin time, where applicable) and close surveillance for bleeding events; argatroban was withheld, reduced, or discontinued in the presence of suspected ICH, major extracranial bleeding, or other predefined safety triggers.

### 2.3. Data collection

Data were extracted retrospectively from the hospital electronic medical record system and the institutional stroke registry using a standardized case report form. The following domains were collected: demographics and baseline characteristics, including age, sex, body mass index, vascular risk factors (hypertension, diabetes mellitus, atrial fibrillation, coronary artery disease), lifestyle exposures (current smoking and alcohol use), and prior cerebrovascular history (prior stroke/transient ischemic attack [TIA]); time metrics and clinical severity, including symptom onset time (or last known well), arrival time, onset-to-needle time, baseline NIHSS score, and admission physiologic parameters (systolic/diastolic blood pressure and blood glucose); imaging and etiologic classification, including baseline non-contrast CT/MRI findings, Alberta Stroke Program Early CT Score, when applicable, vascular imaging results (computed tomography angiography [CTA]/magnetic resonance angiography [MRA]) with documentation of large vessel occlusion, and stroke subtype according to the Trial of ORG 10172 in Acute Stroke Treatment criteria; and treatment-related variables, including thrombolytic administration (dose, timing, and completion time), argatroban initiation time and duration, laboratory monitoring parameters, and concomitant therapies.

Outcome data included early neurological response (NIHSS at 24 hours and day 7, and derived ΔNIHSS and responder status), functional outcomes at 90 days (modified Rankin Scale [mRS] score obtained from outpatient visits or structured telephone follow-up), in-hospital outcomes (length of stay, discharge NIHSS, discharge disposition), and imaging outcomes (recanalization status on follow-up CTA/MRA, when performed, infarct growth/volume change based on follow-up CT/MRI according to routine clinical timing). Safety data captured ICH on follow-up neuroimaging (symptomatic ICH [sICH] and hemorrhage subtypes, including parenchymal hematoma [PH]), extracranial bleeding events (gastrointestinal, urinary tract, and puncture-site bleeding), transfusion requirement, and other adverse events (allergic reactions, hepatic enzyme elevation, thrombocytopenia), as well as in-hospital and 90-day all-cause mortality. Imaging outcomes were obtained as part of routine care rather than according to a fixed protocolized schedule; therefore, the modality and timing of follow-up CT, MRI, CTA, or MRA could vary among patients. To enhance data quality, 2 investigators independently reviewed key variables and outcomes; discrepancies were resolved by consensus with a senior clinician, and missing or implausible values were cross-checked against source documentation (progress notes, medication administration records, laboratory reports, and imaging reports).

To reduce potential bias from heterogeneous imaging assessment, imaging information was cross-checked against radiology reports and original source documentation when available. Recanalization and infarct growth results were, therefore, interpreted cautiously as routine-care imaging endpoints rather than centrally adjudicated trial endpoints.

### 2.4. Statistical analysis

All statistical analyses were performed using IBM SPSS Statistics, version 26.0 (IBM Corp., Armonk). Continuous variables were assessed for distributional characteristics and are presented as mean ± standard deviation for approximately normally distributed data or as median with interquartile range (IQR) for non-normally distributed data. Between-group comparisons for continuous variables were conducted using the independent-samples *t* test for normally distributed variables and the Mann–Whitney *U* test for non-normally distributed variables. Categorical variables are reported as number (percentage) and were compared using the Pearson chi-square test; the Fisher exact test was applied when expected cell counts were small. For ordinal outcomes, the mRS distribution was compared between groups using a nonparametric rank-based approach. All statistical tests were two-sided, and a *P* value < .05 was considered statistically significant.

## 3. Results

### 3.1. Baseline demographic and clinical characteristics

A total of 125 patients were included, with 67 in the control group and 58 in the observation group. Demographic variables were comparable between groups, including age (63.5 ± 9.0 vs 66.2 ± 10.6 years; *t* = −1.54, *P* = .127), sex distribution (male: 59.7% vs 51.7%; χ^2^ = 0.80, *P* = .370), and body mass index (24.2 ± 2.9 vs 24.6 ± 3.4 kg/m^2^; *t* = −0.62, *P* = .538). The prevalence of major vascular risk factors and medical history variables did not differ significantly, including hypertension, diabetes mellitus, atrial fibrillation, coronary artery disease, current smoking, current alcohol use, and prior stroke/TIA (all *P* > .05). Admission stroke severity and time-window indices were also balanced, with similar baseline NIHSS scores (median, 9.0 [IQR, 7.0–12.5] vs 9.0 [6.2–11.8]; *Z* = 0.39, *P* = .695) and onset-to-needle time (median, 138.7 [116.8–174.5] vs 139.5 [122.1–166.8] min; *Z* = 0.00, *P* = .998). Admission blood pressure and glucose levels were comparable. Imaging markers, including the large vessel occlusion rate and the Alberta Stroke Program Early CT Score, showed no significant differences, and the overall Trial of ORG 10172 in Acute Stroke Treatment subtype distribution was similar (χ^2^ = 7.97, *P* = .093; Table 1).

**Table 1:** Baseline characteristics between groups.

Variable	Control (n = 67)	Observation (n = 58)	Test statistic	*P* value
Demographics				
Age, yr	63.5 ± 9.0	66.2 ± 10.6	*t* = −1.54	.127
Male sex, n (%)	40 (59.7)	30 (51.7)	χ^2^ = 0.80	.370
BMI, kg/m^2^	24.2 ± 2.9	24.6 ± 3.4	*t* = −0.62	.538
Vascular risk factors and medical history				
Hypertension, n (%)	41 (61.2)	32 (55.2)	χ^2^ = 0.46	.496
Diabetes mellitus, n (%)	12 (17.9)	11 (19.0)	χ^2^ = 0.02	.879
Atrial fibrillation, n (%)	12 (17.9)	8 (13.8)	χ^2^ = 0.17	.684
Coronary artery disease, n (%)	7 (10.4)	12 (20.7)	χ^2^ = 1.81	.179
Current smoking, n (%)	23 (34.3)	23 (39.7)	χ^2^ = 0.17	.679
Current alcohol use, n (%)	11 (16.4)	14 (24.1)	χ^2^ = 0.76	.385
Prior stroke/TIA, n (%)	11 (16.4)	7 (12.1)	χ^2^ = 0.24	.624
Admission stroke severity and time-window–related indices				
Baseline NIHSS score, median (IQR)	9.0 (7.0–12.5)	9.0 (6.2–11.8)	*Z* = 0.39	.695
Onset-to-needle time (OTT), min, median (IQR)	138.7 (116.8–174.5)	139.5 (122.1–166.8)	*Z* = 0.00	.998
Systolic blood pressure, mm Hg	154.3 ± 19.3	151.7 ± 19.9	*t* = 0.73	.468
Diastolic blood pressure, mm Hg	90.4 ± 10.8	89.8 ± 10.8	*t* = 0.28	.780
Admission glucose, mmol/L, median (IQR)	7.9 (6.8–8.8)	7.5 (6.3–8.7)	*Z* = 1.61	.107
Imaging and etiologic classification				
Large vessel occlusion on CTA/MRA, n (%)	28 (41.8)	25 (43.1)	χ^2^ = 0.02	.882
ASPECTS, median (IQR)	9.0 (8.0–9.0)	9.0 (8.0–9.0)	*Z* = 0.35	.713
TOAST – LAA, n (%)	28 (41.8)	15 (25.9)	–	–
TOAST – CE, n (%)	20 (29.9)	18 (31.0)	–	–
TOAST – SVO, n (%)	11 (16.4)	9 (15.5)	–	–
TOAST – other, n (%)	7 (10.4)	13 (22.4)	–	–
TOAST – undetermined, n (%)	1 (1.5)	4 (6.9)	–	–
TOAST etiology (overall)	–	–	χ^2^ = 7.97	.093

ASPECTS = Alberta Stroke Program Early CT Score, BMI = body mass index, CE = cardioembolism, CTA = computed tomography angiography, LAA = large-artery atherosclerosis, MRA = magnetic resonance angiography, NIHSS = National Institutes of Health Stroke Scale, SVO = small-vessel occlusion, TIA = transient ischemic attack, TOAST = Trial of ORG 10172 in Acute Stroke Treatment.

### 3.2. Primary efficacy outcomes

Compared with the control group, the observation group demonstrated significantly greater early neurological improvement, with larger ΔNIHSS at 24 hours (median, 4.0 vs 3.0; *Z* = −3.25, *P* = .001) and ΔNIHSS at 7 days (median, 6.0 vs 4.0; *Z* = −3.45, *P* < .001). The proportion achieving a clinically relevant improvement (NIHSS decrease ≥4) was higher in the observation group both at 24 hours (67.2% vs 41.8%; χ^2^ = 8.10, *P* = .004) and 7 days (81.0% vs 59.7%; χ^2^ = 6.69, *P* = .010). At 90 days, functional outcomes favored the observation group, including higher rates of mRS 0 to 1 (69.0% vs 47.8%; χ^2^ = 5.72, *P* = .017) and mRS 0 to 2 (87.9% vs 70.1%; χ^2^ = 5.80, *P* = .016), with an overall improvement in the ordinal mRS distribution (shift analysis: *Z* = 3.13, *P* = .001). Imaging-related indices were also more favorable in the observation group, showing a higher recanalization rate (74.1% vs 55.2%; χ^2^ = 4.83, *P* = .028) and lower infarct growth (median 20.7 vs 28.2 mL; *Z* = 3.21, *P* = .001). The incidence of END was lower in the observation group (1.7% vs 11.9%; Fisher exact, *P* = .037; Table 2).

**Table 2:** Primary efficacy outcomes, imaging/recanalization indices, and early neurological deterioration.

Outcome	Control (n = 67)	Observation (n = 58)	Test statistic	*P* value
Early neurological improvement				
ΔNIHSS at 24 h, median (IQR)	3.0 (1.0–4.0)	4.0 (3.0–6.0)	*Z* = −3.25	.001
NIHSS decrease ≥4 at 24 h, n (%)	28 (41.8)	39 (67.2)	χ^2^ = 8.10	.004
ΔNIHSS at 7 d, median (IQR)	4.0 (3.0–6.0)	6.0 (4.3–8.0)	*Z* = −3.45	<.001
NIHSS decrease ≥4 at 7 d, n (%)	40 (59.7)	47 (81.0)	χ^2^ = 6.69	.010
Functional outcome at 90 d				
mRS 0–1, n (%)	32 (47.8)	40 (69.0)	χ^2^ = 5.72	.017
mRS 0–2, n (%)	47 (70.1)	51 (87.9)	χ^2^ = 5.80	.016
mRS (shift analysis), median (IQR)	2.0 (1.0–3.0)	0.0 (0.0–2.0)	*Z* = 3.13	.001
Imaging/recanalization indices				
Recanalization on CTA/MRA, n (%)	37 (55.2)	43 (74.1)	χ^2^ = 4.83	.028
Infarct growth, mL, median (IQR)	28.2 (20.3–40.5)	20.7 (12.1–32.1)	*Z* = 3.21	.001
Safety-related neurological worsening				
Early neurological deterioration (END), n (%)	8 (11.9)	1 (1.7)	Fisher exact	.037

CTA = computed tomography angiography, IQR = interquartile range, MRA = magnetic resonance angiography, mRS = modified Rankin Scale, NIHSS = National Institutes of Health Stroke Scale, ΔNIHSS = change from baseline NIHSS (baseline − follow-up NIHSS).

### 3.3. Secondary outcomes

The observation group was associated with a shorter length of hospital stay compared with the control group (median, 8.0 [IQR 7.0–10.8] vs 10.0 [IQR, 9.0–13.0] days; *Z* = 3.24, *P* = .001). Neurological status at discharge differed between groups, with a lower discharge NIHSS in the observation group (median, 3.0 [2.0–5.0] vs 5.0 [3.0–6.0]; *Z* = 3.29, *P* < .001). Discharge disposition distributions were numerically more favorable in the observation group (home discharge, 74.1% vs 65.7%), but the overall 3-category comparison did not reach statistical significance (χ^2^ = 2.57, *P* = .277). During the 90-day follow-up, the composite rate of recurrent stroke or TIA was lower in the observation group (5.2% vs 20.9%; Fisher exact, *P* = .017), whereas recurrent ischemic stroke and TIA, analyzed separately, showed nonsignificant between-group differences (both *P* > .05; Table 3).

**Table 3:** Secondary efficacy outcomes (in-hospital outcomes and 90-day cerebrovascular events).

Outcome	Control (n = 67)	Observation (n = 58)	Test statistic	*P* value
Hospital-related outcomes				
Length of hospital stay, d, median (IQR)	10.0 (9.0–13.0)	8.0 (7.0–10.8)	*Z* = 3.24	.001
Discharge NIHSS, median (IQR)	5.0 (3.0–6.0)	3.0 (2.0–5.0)	*Z* = 3.29	<.001
Discharge destination, n (%)			χ^2^ = 2.57	.277
Home	44 (65.7)	43 (74.1)	–	–
Inpatient rehabilitation/transfer	23 (34.3)	14 (24.1)	–	–
In-hospital death	0 (0.0)	1 (1.7)	–	–
Recurrent events within 90 d				
Recurrent ischemic stroke, n (%)	9 (13.4)	2 (3.4)	Fisher exact	.061
Transient ischemic attack, n (%)	5 (7.5)	1 (1.7)	Fisher exact	.215
Recurrent stroke or TIA (composite), n (%)	14 (20.9)	3 (5.2)	Fisher exact	.017

IQR = interquartile range, NIHSS = National Institutes of Health Stroke Scale, TIA = transient ischemic attack.

### 3.4. Safety outcomes

Overall, hemorrhagic and nonhemorrhagic adverse events were uncommon in both groups. The incidence of sICH was numerically lower in the observation group than in the control group (1.7% vs 6.0%), but the between-group difference was not statistically significant (Fisher exact, *P* = .371). Any ICH was observed in 11.9% of control patients and 6.9% of observation patients (χ^2^ = 0.91, *P* = .340), and PH1/PH2 events were rare, with no significant between-group differences (both *P* > .05). Rates of extracranial bleeding (gastrointestinal, urinary tract, and puncture-site bleeding) and transfusion requirement were low and comparable between groups (all *P* > .05). Other adverse events, including allergic reactions, hepatic enzyme elevation, and thrombocytopenia, did not differ significantly (all *P* > .05). No in-hospital deaths occurred in either group, and 90-day all-cause mortality was rare, with a single death recorded in the observation group (1.7% vs 0.0%; Fisher exact, *P* = .464; Table 4).

**Table 4:** Safety outcomes (intracranial hemorrhage, extracranial bleeding, other adverse events, and mortality).

Outcome	Control (n = 67)	Observation (n = 58)	Test statistic	*P* value
Intracranial hemorrhage–related outcomes				
Symptomatic ICH (sICH), n (%)	4 (6.0)	1 (1.7)	Fisher exact	.371
Any ICH, n (%)	8 (11.9)	4 (6.9)	χ^2^ = 0.91	.340
PH1, n (%)	2 (3.0)	1 (1.7)	Fisher exact	1.000
PH2, n (%)	2 (3.0)	0 (0.0)	Fisher exact	.499
Extracranial bleeding and transfusion				
Gastrointestinal bleeding, n (%)	3 (4.5)	1 (1.7)	Fisher exact	.623
Urinary tract bleeding, n (%)	2 (3.0)	1 (1.7)	Fisher exact	1.000
Puncture-site bleeding/hematoma, n (%)	4 (6.0)	2 (3.4)	Fisher exact	.685
Transfusion requirement, n (%)	1 (1.5)	0 (0.0)	Fisher exact	1.000
Other adverse events				
Allergic reaction, n (%)	1 (1.5)	0 (0.0)	Fisher exact	1.000
Hepatic enzyme elevation, n (%)	5 (7.5)	3 (5.2)	χ^2^ = 0.27	.602
Thrombocytopenia, n (%)	2 (3.0)	1 (1.7)	Fisher exact	1.000
Mortality				
In-hospital all-cause death, n (%)	0 (0.0)	0 (0.0)	–	–
90-d all-cause mortality, n (%)	0 (0.0)	1 (1.7)	Fisher exact	.464

ICH = intracranial hemorrhage, PH = parenchymal hematoma (ECASS classification).

## 4. Discussion

In this single-center retrospective cohort study evaluating thrombolysis combined with argatroban in patients with AIS, the combination strategy was associated with greater early neurological improvement and more favorable functional recovery compared with standard thrombolysis alone. Because patients were not randomly assigned to treatment, these findings should be interpreted as observed associations rather than evidence that the combination strategy caused the observed outcomes. Specifically, the observation group had a larger reduction in NIHSS within the first 24 hours and by day 7, accompanied by a higher proportion of patients meeting a clinically meaningful early improvement threshold. These early differences were paralleled by higher rates of favorable 90-day mRS categories and a shift toward lower disability across the ordinal mRS distribution. In addition, imaging-related indices suggested a higher likelihood of arterial recanalization and less infarct growth, while END occurred less frequently in the combination group. Collectively, these associations support further prospective evaluation of whether adding a targeted anticoagulant effect to thrombolysis can reduce early thrombus propagation and downstream microthrombotic processes that may contribute to neurological worsening and incomplete tissue-level reperfusion.

From a pathophysiological perspective, tenecteplase offers pharmacological advantages, including increased fibrin specificity and a longer half-life that permits single-bolus administration, thereby potentially reducing treatment delays and improving delivery consistency in routine practice.^[11,12]^ Nonetheless, even with successful clot lysis, thrombin generation and platelet activation can persist and may promote reocclusion, incomplete reperfusion, and early infarct expansion. Argatroban, as a direct thrombin inhibitor, may mitigate these processes by inhibiting thrombin-mediated fibrin formation and thrombus stabilization, theoretically complementing the fibrinolytic effect. The lower observed rates of END and infarct growth in the combination group are compatible with this mechanistic rationale, but they do not establish causality because of the nonrandomized design and possible residual confounding.

Contemporary randomized evidence has established that tenecteplase (0.25 mg/kg) is a clinically acceptable alternative to alteplase for intravenous thrombolysis in eligible AIS populations, with comparable functional outcomes and safety. In the ORIGINAL noninferiority randomized clinical trial, tenecteplase achieved similar 90-day excellent functional outcomes (mRS 0–1) compared with alteplase and a similar safety profile, supporting its use in routine thrombolysis pathways.^[11,13]^ These data align with the European Stroke Organisation expedited recommendation, which endorses tenecteplase 0.25 mg/kg as a safe and effective alternative to alteplase and recommends against higher-dose tenecteplase (0.40 mg/kg) because of less favorable evidence.^[12,14]^ Recent meta-analytic syntheses have similarly suggested noninferior safety and potentially favorable efficacy signals for tenecteplase – particularly at 0.25 mg/kg – though heterogeneity across trials and populations remains.^[15]^

In contrast, evidence for adjunctive argatroban with thrombolysis has been mixed. The ARAIS randomized clinical trial (alteplase ± argatroban) did not demonstrate a statistically significant improvement in excellent 90-day neurological function with the routine addition of argatroban to alteplase, indicating that the benefit may not be uniform across unselected thrombolysis candidates.^[16]^ More recently, the multiarm optimization of stroke thrombolysis phase 3 trial evaluated adjunctive argatroban or eptifibatide added to intravenous thrombolysis (including both alteplase and tenecteplase) and did not show improvement in functional outcomes, emphasizing the challenge of identifying an add-on strategy that improves disability in broad AIS cohorts.^[17]^ However, a randomized trial focused on patients with END reported improved 90-day functional outcomes with argatroban plus antiplatelet therapy compared with antiplatelet therapy alone, suggesting that argatroban may confer benefit in a clinically enriched subgroup characterized by early progression.^[10,18]^ Against this background, the present study’s findings – showing concurrent improvements in early neurological response, recanalization-related indices, and disability – are compatible with a selection-sensitive effect and underscore the importance of treatment timing, patient phenotype, and concomitant reperfusion modality when interpreting the therapeutic value of adjunctive anticoagulation.

If validated, tenecteplase combined with argatroban may represent a pragmatic strategy to enhance early neurological recovery and functional independence in selected AIS patients, particularly where early progression or thrombus instability is suspected. Implementation should emphasize strict eligibility screening, repeat neuroimaging when clinically indicated, and standardized post-thrombolysis monitoring for hemorrhagic transformation. Initiation of argatroban should be protocolized (e.g., after completion of thrombolysis and confirmation of clinical stability), with activated partial thromboplastin time-guided titration and predefined stopping rules for bleeding or neurological deterioration. These measures may help balance potential efficacy gains against hemorrhagic risk. This study has several strengths. First, it reflects a real-world cohort with consecutive enrollment across a contemporary period, enhancing clinical relevance. Second, efficacy assessment captured both early neurological endpoints (ΔNIHSS and responder proportions) and 90-day disability outcomes, which are widely accepted and clinically interpretable. Third, incorporation of imaging-related indices (recanalization and infarct growth) supports biological plausibility by linking treatment exposure to mechanistically relevant intermediate outcomes. Finally, safety evaluation included ICH subtyping and extracranial bleeding events, enabling a balanced appraisal of benefit and risk.

Important limitations should be acknowledged. The retrospective, nonrandomized design introduces the potential for selection bias, confounding by indication, and residual imbalance in unmeasured variables (e.g., clot composition, collateral status, nuanced workflow delays, collateral circulation, or physician preference). Although measured baseline characteristics were comparable between groups, comparability of recorded variables cannot eliminate residual or unmeasured confounding, and treatment selection may have been influenced by perceived bleeding risk, stroke phenotype, vessel status, or evolving institutional practice. The single-center setting and moderate sample size limit generalizability and reduce power for rare safety events, particularly sICH and mortality. Outcome ascertainment may be influenced by variable follow-up completeness or rehabilitation access. Imaging endpoints are another important source of potential bias: follow-up CT/MRI/CTA/MRA was performed according to routine clinical indications rather than a uniform study schedule, and differences in modality, timing, and image quality could affect ascertainment of recanalization and infarct volume change. Although 2 investigators reviewed key imaging-related variables and discrepancies were resolved by consensus, the absence of a blinded core imaging laboratory and standardized imaging time points may have introduced detection bias and measurement variability. Additionally, treatment adherence and argatroban dosing/titration could vary in routine practice. These constraints preclude causal inference; prospective multicenter randomized trials and carefully designed observational studies with robust adjustment (e.g., propensity-weighted analyses and standardized imaging schedules) are required.

## 5. Conclusion

In this single-center retrospective cohort of 125 patients with AIS, baseline characteristics were comparable between groups. Compared with standard thrombolysis alone, thrombolysis combined with argatroban was associated with greater early neurological improvement, higher rates of favorable 90-day functional outcomes, higher recanalization rates, reduced infarct growth, and fewer END events. Secondary outcomes were also associated with the combination strategy, including shorter hospitalization and lower discharge NIHSS scores. Safety events were uncommon, with no significant increase in hemorrhagic complications or other adverse events. Because treatment allocation was nonrandomized, these findings should be considered hypothesis-generating and require confirmation in prospective randomized or rigorously adjusted multicenter studies.

## Author contributions

**Conceptualization:** Shaoyue Huang, Xiaoxuan Wang, Dongxu Li, Xiaowei Qiu, Zhen Hong.

**Data curation:** Shaoyue Huang, Xiaoxuan Wang, Dongxu Li, Xiaowei Qiu, Zhen Hong.

**Formal analysis:** Shaoyue Huang, Xiaoxuan Wang, Dongxu Li, Xiaowei Qiu, Zhen Hong.

**Funding acquisition:** Shaoyue Huang, Zhen Hong.

**Investigation:** Zhen Hong.

**Writing – original draft:** Shaoyue Huang, Zhen Hong.

**Writing – review & editing:** Shaoyue Huang, Zhen Hong.
